# Blood Transfusion Requirements for Patients With Sarcomas Undergoing Combined Radio- and Chemotherapy

**DOI:** 10.1080/13577140500180005

**Published:** 2005

**Authors:** Karen R. Sherbourne, Helena M. Earl, Lynne Whitehead, Sarah J. Jefferies, Neil G. Burnet

**Affiliations:** 1 Oncology Centre Addenbrooke's Hospital Hills Road Cambridge CB2 2QQ UK; 2 University of Cambridge Department of Oncology Oncology Centre Addenbrooke's Hospital Hills Road Cambridge CB2 2QQ UK; 3 Pharmacy Department Addenbrooke's Hospital Hills Road Cambridge CB2 2QQ UK

## Abstract

Patients with bony and soft tissue sarcomas may require intensive treatment with chemotherapy and radiotherapy, which often leads to a fall in haemoglobin levels, requiring blood transfusion. There may be advantages in predicting which patients will require transfusion, partly because anaemia and hypoxia may worsen the response of tumours to chemotherapy and radiotherapy. Between 1997 and 2003, a total of 26 patients who received intensive treatment with curative intent were identified. Transfusions were given to maintain the haemoglobin at 10g/dl or above during chemotherapy, and at 12 g/dl or above during radiotherapy. Eighteen (69%) required a transfusion, the majority as a result of both the chemotherapy and RT criteria. There were 78 transfusion episodes, and 181 units of blood given. In the 18 patients who required transfusion, the average number of units was 10.1, but seven patients required more blood than this. The most significant factor influencing blood transfusion was choice of intensive chemotherapy. Intensive chemotherapy and presenting Hb less than 11.6 g/dl identified 13 out of 18 patients who needed transfusion. Adding a drop in haemoglobin of greater than 1.7 g/dl after one cycle of chemotherapy identified 16 out of 18 patients who required transfusion. The seven patients who had heavy transfusion requirements were identified by age 32 or less, intensive chemotherapy and a presenting Hb of 12 g/dl or less. Erythropoietin might be a useful alternative to transfusion in selected patient groups, especially those with heavy transfusion requirements.


*Sarcoma*, September/December 2005; 9(3/4): 119–125

Taylor & Francis

Taylor & Francis Group

**ORIGINAL ARTICLE**

### Blood transfusion requirements for patients with sarcomas undergoing combined radio- and chemotherapy

KAREN R. SHERBOURNE[1], HELENA M. EARL[1,2], LYNNE WHITEHEAD[3], SARAH J. JEFFERIES[1], & NEIL G. BURNET[1,2]

[1]*Oncology Centre, Addenbrooke's Hospital, Hills Road, Cambridge CB2 2QQ, UK,* [2]*University of Cambridge Department of Oncology, Oncology Centre, Addenbrooke's Hospital, Hills Road, Cambridge CB2 2QQ, UK, and* [3]*Pharmacy Department, Addenbrooke's Hospital, Hills Road, Cambridge CB2 2QQ, UK*

**Abstract**
Patients with bony and soft tissue sarcomas may require intensive treatment with chemotherapy and radiotherapy, which often leads to a fall in haemoglobin levels, requiring blood transfusion. There may be advantages in predicting which patients will require transfusion, partly because anaemia and hypoxia may worsen the response of tumours to chemotherapy and radiotherapy. Between 1997 and 2003, a total of 26 patients who received intensive treatment with curative intent were identified. Transfusions were given to maintain the haemoglobin at 10g/dl or above during chemotherapy, and at 12 g/dl or above during radiotherapy. Eighteen (69%) required a transfusion, the majority as a result of both the chemotherapy and RT criteria. There were 78 transfusion episodes, and 181 units of blood given. In the 18 patients who required transfusion, the average number of units was 10.1, but seven patients required more blood than this. The most significant factor influencing blood transfusion was choice of intensive chemotherapy. Intensive chemotherapy and presenting Hb less than 11.6 g/dl identified 13 out of 18 patients who needed transfusion. Adding a drop in haemoglobin of greater than 1.7 g/dl after one cycle of chemotherapy identified 16 out of 18 patients who required transfusion. The seven patients who had heavy transfusion requirements were identified by age 32 or less, intensive chemotherapy and a presenting Hb of 12 g/dl or less. Erythropoietin might be a useful alternative to transfusion in selected patient groups, especially those with heavy transfusion requirements.

**Keywords:** *Sarcoma, chemotherapy, radiotherapy, blood transfusion, erythropoietin*

### Introduction

Patients with bony and soft tissue sarcomas may require intensive treatment with chemotherapy and radiotherapy, in addition to surgery. Myelosuppressive chemotherapy frequently leads to a fall in haemoglobin levels, requiring blood transfusion. It is our policy to maintain the haemoglobin greater than 10 g/dl for patients receiving chemotherapy, in order to avoid symptoms of anaemia with consequent reduction in activity and quality of life [1–3]. There is also the possibility that anaemia may impair tumour response to chemotherapy [4–8]. In addition, radiotherapy is known to be more effective in the presence of oxygen [9,10], and patients with lower haemoglobin levels have been shown, in some tumour types, to have lower rates of local tumour control [11–15]. It is therefore our policy to maintain haemoglobin levels greater than 12 g/dl for the duration of radiotherapy.

The proportion of patients requiring transfusion is difficult to establish, though it obviously depends upon the tumour types included in any series. For some patients who are identified as requiring transfusion, it may be difficult to schedule transfusion, particularly when this must precede radiotherapy treatment. It is particularly problematic for patients travelling long distances to their Sarcoma Specialist Centre. The possibility of predicting which patients would require transfusion would therefore be attractive.

Correspondence: K. R. Sherbourne, Oncology Centre, Addenbrooke's Hospital, Hills Road, Cambridge CB2 2QQ, UK. E-mail: Karen.sherbourne@addenbrookes.nhs.uk

ISSN 1357–714X print/ISSN 1369–1643 online/05/(03-04)0119-7 © 2005 Taylor & Francis
DOI: 10.1080/13577140500180005

This might also offer the possibility of preventative treatment, for example with recombinant erythropoietin (EPO), with advantages to the patient and the Blood Transfusion Service.

We therefore examined the transfusion needs of our patients requiring intensive chemo- and radiotherapy as part of their sarcoma management.

### Patients and methods

Patients having combination chemotherapy and radiotherapy (RT) were identified retrospectively from our database. All patients receiving radical treatment between 1997 and 2003 were included, and a total of 26 patients were identified. The diagnoses were classified into three categories, firstly PNET (including Ewing's sarcoma), secondly osteo- and chondrosarcoma, and finally all other sarcomas (Table I). There were 15 men and 11 women. The mean age of the patient population was 35.5 years (median 31.0) and the ages ranged from 16 to 70 years (Figure 1). One additional patient received radiotherapy using a brachytherapy implant inserted at the time of re-operation prior to chemotherapy, and was excluded from the study because of the timing of the radiotherapy treatment.

The chemotherapy schedules varied according to clinical indications and protocol changes during the study period (Table II). Patients received chemotherapy and radiotherapy in one of three ways (Table III): nine patients received 'sandwich' treatment with chemotherapy stopping for the duration of the RT and restarting afterwards; five received RT and chemotherapy concurrently, using a schedule based on the EICESS 92 and EuroEwings 99, with the radiotherapy starting after a minimum of two cycles of chemotherapy; 12 patients received radiotherapy after completion of chemotherapy. In one of these 12 cases the intention had been to continue chemotherapy after radiotherapy, but the patient was unable to tolerate further chemotherapy due to uncontrollable nausea.

Blood transfusion requirements were noted, including dates of transfusion and the total number of units. The timing of transfusions was correlated with the timing of the chemotherapy and radiotherapy for the patient. Blood transfusions given as

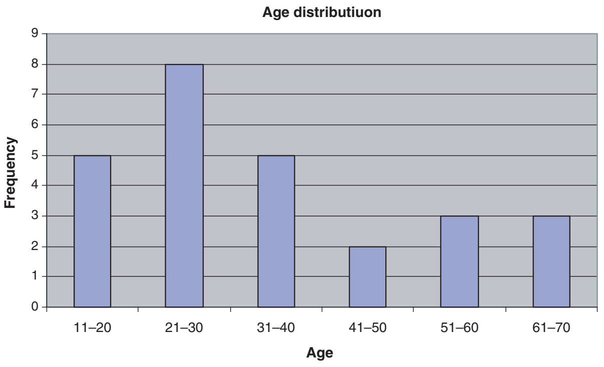

Figure 1. Age distribution of patients.

Table I. Types of sarcoma treated.

| Tumour group categories | Number of patients | Total |
|---|---|---|
| PNET/Ewing's | 5 | 5 |
| Osteosarcoma/ chondrosarcoma | | |
| Osteosarcoma | 4 | |
| Chondrosarcoma | 1 | 5 |
| Soft tissue | | |
| Embryonal rhabdosarcoma | 2 | |
| Alveolar rhabdosarcoma | 2 | |
| Fibrosarcoma | 1 | |
| Alveolar soft part sarcoma | 1 | |
| Liposarcoma | 1 | |
| Leiomyosarcoma | 2 | |
| Epitheliod sarcoma | 1 | |
| Synovial sarcoma | 3 | |
| Malignant schwannoma | 1 | |
| Malignant peripheral nerve sheath tumour | 1 | |
| NOS (not otherwise specified) | 1 | 16 |
| Total | | 26 |

Table II. Chemotherapy protocols used.

| Protocol | Number of patients |
|---|---|
| MMT 98: CEV/IVA/Cyclo/Etop/Carbo/VAC (carboplatin, epirubicin, vincristine/ifosfamide, vincristine, actinomycin D/ cyclophosphamide/etoposide/ carboplatin/vincristine, actinomycin D, cyclophosphamide | 1 |
| PD (*cis*-platinum & doxorubicin) | 4 |
| VIDE (vincristine, ifosfamide doxorubicin and etoposide) | 3 |
| EICESS 92: EVAIA (etoposide, vincristine, doxorubicin, ifosfamide and actinomycin D) | 3 |
| EuroEwing 99: VIDE/VIA (vincristine, ifosfamide, doxorubicin & etoposide/vincristine, ifosfamide & actinomycin D) | 2 |
| VID (vincristine, ifosfamide and doxorubicin) | 2 |
| ID (ifosfamide & doxorubicin) | 7 |
| VIE (vincristine, ifosfamide and etoposide) | 1 |
| Carbo D (carboplatin & doxorubicin) | 1 |
| D (doxorubicin) | 2 |

Table III. Timing of radiotherapy and chemotherapy.

| Timing of RT and ChT | Number of patients |
|---|---|
| 'Sandwich'* | 9 |
| Concurrent¶ | 5 |
| RT given after chemotherapy | 12 |

*Chemotherapy–RT–chemotherapy, one treatment following the previous, with no overlap. ¶RT and chemotherapy given on same day.

Table IV. Timing of transfusions required.

| Timing of transfusions | Number of patients | Total |
|---|---|---|
| Transfusion required | | |
| Only during RT | 1 | |
| Only during ChT | 6 | |
| During both RT and ChT | 11 | 18 (69%) |
| No transfusion required | 8 (31%) | |

Transfusion criteria were: for RT maintain Hb >12 g/dl, and for chemotherapy maintain Hb >10 g/dl. See text for further details.

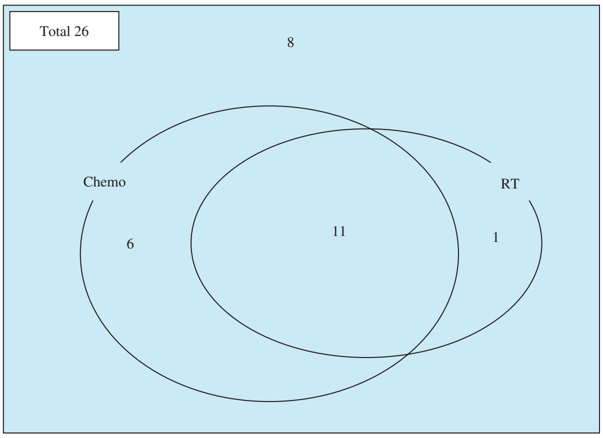

Figure 2. Venn diagram illustrating the transfusion requirements in the study group of 26 patients.

a result of surgery are not included, because these are so specifically related to site and surgical techniques. Transfusions were given for two main reasons: firstly, to maintain the haemoglobin at 10 g/dl or above during chemotherapy, and secondly, to raise the haemoglobin level to 12 g/dl or above prior to and during radiotherapy.

### Results

Of the 26 patients in our series, 18 (69%) required a transfusion during treatment (Table IV). The majority required treatment as the result of both the chemotherapy and RT criteria. Twelve patients (46%) required transfusion for RT: in 11 cases, the patients had already required transfusion because of low haemoglobin during the preceding chemotherapy (Figure 2), and in only one was transfusion required for RT alone. In this patient, the haemoglobin level had remained stable and over 10 g/dl during chemotherapy but was below 12 g/dl at the start of RT.

There were 78 transfusion episodes, and 181 units of blood given. Figure 3 shows the transfusion requirements of the group. The average number of transfused units of blood was 7.0 (standard deviation (SD 7.2), and the average number of transfusion episodes was 3.0 per patient (SD 3.1).

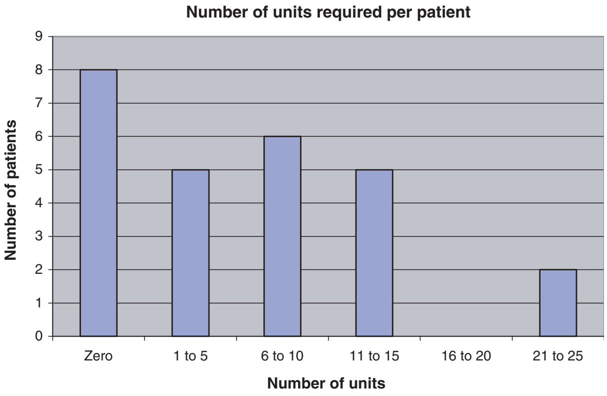

Figure 3. Frequency distribution of number of units of blood required per patient.

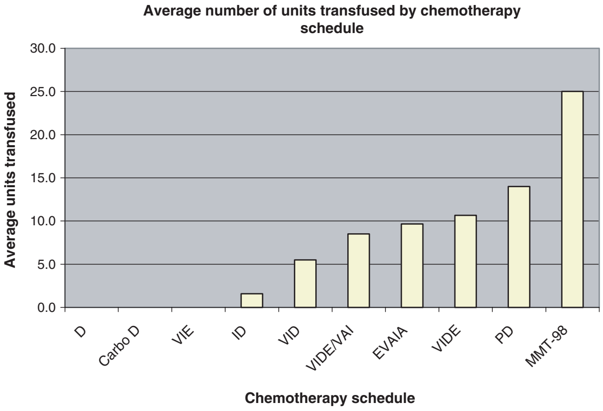

Figure 4. Frequency distribution of number of units of blood required according to chemotherapy protocol. The abbreviations are explained in Table II.

Considering only the 18 patients who required transfusion, the average number of units required was 10.1, and the average number of transfusion episodes was 4.3. A heavy transfusion requirement was defined as more than 10 units of blood.

There was clear evidence of variation in transfusion need according to chemotherapy schedule, with more intensive protocols requiring larger amounts of blood (Figure 4). Of the 26 patients, five received chemotherapy lasting more than 18 weeks (the equivalent of six cycles delivered 3-weekly); four of these had heavy transfusion requirements, of more than 10 units of blood. In two patients, a total of 24 and 25 units were required to maintain haemoglobin at the appropriate levels. The patient who required 24 units of blood had an osteosarcoma treated with *cis*-platinum and doxorubicin; there was no apparent reason for this patient tolerating chemotherapy less well than expected, though she was 32 at the time of treatment. The 25-unit transfusion was required by a patient with embryonal rhabdomyosarcoma, who received high-dose therapy with peripheral stem cell rescue. In addition to these two patients, five others received greater than 10 units of blood: three received *cis*-platinum as part of their chemotherapy.

There was no relationship between age at presentation and the need for transfusion, or the number of units transfused. However, all the patients who required heavy transfusion were younger, 32 or less. There was no exact relationship between sex and transfusion requirement, either in general or amongst the heavy transfusion group (more than 10 units of blood). Although more units of blood were required by female patients (mean 9.6 units per patient) than men (mean 5.0 units), this was not statistically significant ($P \approx 0.1$). However, the female patients on average had lower presenting haemoglobin (mean 11.9 g/dl, range 9.2–13.9 g/dl) than the men (mean 13.5 g/dl, range 10.5–15.2 g/dl).

All seven of the heavy transfusion patients had a presenting haemoglobin (Hb) of 12 or less g/dl. Three other patients also had low Hb levels at presentation, two of whom required no transfusion at all. Twelve patients presented with a haemoglobin level of 13 or more g/dl, and eight did require a transfusion, though none had heavy transfusion requirements.

A modest drop in haemoglobin of 1.7 g/dl or less after the first cycle of chemotherapy identified all eight patients who did not require blood. Seven patients who did require blood also had drop in haemoglobin of 1.7 g/dl or less after the first cycle. Haemoglobin drop after two cycles did not add additional discrimination of transfusion needs.

Different combinations of these factors were examined, to assess whether transfusion needs could be predicted. Combining the two factors of intensive chemotherapy (defined as more than three drugs or a regime containing *cis*-platinum) and presenting Hb less than 11.6 g/dl identified 13 out of 18 patients who needed transfusion. Adding the third factor of a drop in haemoglobin of greater than 1.7 g/dl after one cycle of chemotherapy identified 16 out of 18 patients who required some transfusion. Seven patients had heavy transfusion requirements. The combination of age 32 or less, intensive chemotherapy and presenting Hb of 12 g/dl or less identified all seven patients, with no false-positives.

### Discussion

Patients with bony and soft tissue sarcomas frequently require intensive chemotherapy, often with radiotherapy added, as part of a multi-disciplinary management strategy. The intensive nature of the chemotherapy leads to myelosuppression, and anaemia is a frequent complication. We investigated the scale of the transfusion requirements of our patients and found considerable variation in the transfusion needs, which was clearly associated with the intensity of the chemotherapy schedule.

### *Predicting transfusion requirements*

Just over two-thirds of patients required a transfusion at some time during treatment and in these patients the average number of units of blood required was 10.1. A few patients required very large quantities of blood transfusion, and in two, 24 and 25 units were required respectively. However, one-third of patients did not require any transfusion.

Prediction of transfusion needs might help in planning a patient's treatment. This applies particularly to the use of radiotherapy, which is more effective in the presence of adequate tumour oxygenation. In order to achieve the maximum benefit from this treatment modality, it is advisable to transfuse patients at least 48 h prior to radiotherapy, and to maintain haemoglobin levels during the radiotherapy course. Prediction of transfusion needs for chemotherapy is also attractive, since anaemia certainly affects quality of life [1–3]. There are also suggestions that low haemoglobin levels are associated with lower efficacy from chemotherapy [4–8].

The most significant predictor of blood transfusion need was choice of chemotherapy, particularly the use of more than three drugs or a regime containing *cis*-platinum (Figure 4). Intensive chemotherapy and a presenting haemoglobin less than 11.6 g/dl identified 13 out of 18 patients who needed transfusion. Although five other patients who needed transfusion were not identified, these criteria are simple, and available at the time of presentation.

All seven patients who had heavy transfusion requirements were identified by a combination of three factors available at presentation. The heavy transfusion requirement in the younger patients was almost certainly due to the use of more intensive chemotherapy. There was no suggestion that the sex of the patient influenced transfusion needs, except that the average presenting haemoglobin level was lower in women.

### *Anaemia, hypoxia and efficacy of cancer treatment*

Health-related quality of life has been shown to correlate directly with the severity of anaemia in patients with cancer [3,16,17], and raising haemoglobin levels improves quality of life [3,18,19]. Some published work suggests that treatment with erythropoietins has a positive effect on quality of life [1,18,19], but the evidence is inconclusive [20].

Anaemia is known to be an independent prognostic factor for outcome in a number of tumour sites [11,13], including soft tissue sarcoma [15]. Tumour hypoxia is common in many tumour sites, also including soft tissue sarcoma [9,14,15,21,22], where survival is related to tumour oxygenation [15]. Hypoxia may facilitate malignant progression of tumours [4, 10], and there is evidence of increasing metastatic potential in the presence of hypoxia in the laboratory [8] and in patients [21]. It has also been suggested that tumour hypoxia in soft tissue sarcomas is associated with more rapid cell proliferation [22].

As well as the effects on quality of life, it has been suggested that anaemia and hypoxia worsen the response of tumours to chemotherapy [5–7] and radiotherapy [7, 10]. Tumour resistance to radiotherapy in anaemic patients can, at least partially, be prevented or overcome by correction of anaemia, resulting in better locoregional tumour control and overall survival [7]. Although difficult to prove in humans, there is experimental evidence in mice that EPO restores the loss of radiosensivity associated with anaemia [23]. Enhanced radiation sensitivity has been demonstrated in tumours in the laboratory as a result of correction of anaemia resulting from chemotherapy [24].

There is good recent evidence, from double-blind randomised trials, that recombinant human EPO can improve haemoglobin levels in patients having chemotherapy, for both solid [18,25] and haematogenous cancers [26]. Other studies have also shown that EPO can improve haemoglobin levels in chemotherapy-induced anaemia, particularly at levels under or just over 10 g/dl [19,27]. EPO has also been reported to prevent chemotherapy-induced anaemia in patients with primary malignant bone tumours [28]. Treatment with EPO in patients receiving radiotherapy has been shown to increment haemoglobin levels by approximately 0.7 g/dl per week in anaemic patients [12]. The same group have also demonstrated worse outcome associated with anaemia, and a better outcome in those patients whose anaemia reversed rapidly with EPO treatment [12].

Haemoglobin can be maintained continuously using EPO, or, in a stepwise manner, by intermittent transfusion in response to falling haemoglobin levels [29]. The first strategy produces a constant satisfactory haemoglobin level, whilst the second achieves only periodically acceptable levels. Given the concerns that falling haemoglobin might cause increased hypoxia, the maintenance of haemoglobin through the use of erythropoietins has theoretical advantages [29,30–32].

There have been concerns, for many years, that blood transfusion may worsen outcome of patients with cancer, perhaps by an immune suppression mechanism [33,34]. Similar concerns have also been raised regarding EPO treatment in patients with breast [35] and head and neck cancer [36]. In contrast, survival advantage has also been reported in patients treated with EPO [18,37]. It is not entirely clear exactly why these differences in the literature might exist, though there are methodological or data collection issues in some studies. The evidence regarding survival and the use of EPO is inconclusive at present [20].

Blood transfusion is also associated with significant side effects, including risk of administering the wrong blood, infection, anaphylactic shock, iron overload and thrombo-embolism. In one report 20% of all transfusions were associated with side effects [38]. In addition, the future of blood supplies in the UK is threatened by a fall in the donor pool due to an ageing population and increasingly stringent safety criteria [39]. Clearly, prospective trials of both treatments are needed urgently.

### *Costs of erythropoietins for treatment of anaemia*

Using standard doses of erythropoietin, treatment costs approximately £1000 per month. If inadequate response is seen, doses may need to be doubled, which doubles the cost. Attention must also be paid to iron stores, which if low can inhibit response to EPO [17]. By comparison, the cost of transfusion is approximately £400 for a three unit transfusion. A bed cost is difficult to estimate and depends on the specific accounting procedures used.

To look at the relative cost-effectiveness of EPO in different groups of patients, we made a simple cost comparison. In the patients who needed heavy transfusion, there were 49 transfusion episodes. Assuming the cost of blood is £400 per treatment episode, rather than £400 per three unit transfusion, this gives a total cost for this sub-group of £19 600. If this same group were treated with EPO throughout their chemotherapy course, treatment would be for a total of 46.5 patient months. Assuming an approximate cost of £1000 per month, this yields a total cost of £46 500. By this simple calculation EPO would be 2.4 times as expensive as transfusion. In comparison, taking the whole group, there were 78 transfusion episodes, and 136 months of chemotherapy. Using these figures, the relative cost of EPO is 3.2 times the cost of transfusion. Thus, the more cost-effective group to treat with EPO would be those with heavy transfusion requirements.

These calculations are very simple, and do not take into account EPO dose modification that may be necessary, including lowering or increasing dose according to response. Since the cost of transfusion has not included cost of admission to day unit or for overnight stay, the relative difference between EPO and transfusion will be less than these simple calculations indicate. Particularly where severe pressure exists on existing services, where capacity may not be adequate for progressively increasing demand, non-financial considerations may also be warranted. Reduction in the costs of EPO would also alter this balance. In our estimates, which are certainly over simple, EPO is more expensive than blood transfusion by 2.4–3.2 times. In a study in which EPO was actually used for treatment, a higher cost ratio of 3.9 was suggested [41]. In a study of cost-effectiveness of recombinant human EPO (rHuEPO) in the prevention of chemotherapy-induced anaemia, Barosi et al. [40] estimated that the cost of rHuEPO was always greater than $100 000 per quality-adjusted life year (QALY). They concluded that according to current use, rHuEPO is not cost-effective in the treatment of chemotherapy-induced anaemia.

Further work is clearly needed to define more precisely the roles of blood transfusion and EPO treatment, particularly in relation to survival. Reduction in the cost of erythropoietins would clearly facilitate such work. As a result of this study, we propose to follow patients during their chemotherapy and to plan ahead for early blood transfusion, in order to minimise haemoglobin fall. We also propose to study in a prospective manner how EPO might be used as a substitute for blood transfusion.

### Conclusions

The most significant predictor of blood transfusion need was choice of chemotherapy. Intensive chemotherapy and a presenting haemoglobin less than 11.6 g/dl identified 13 out of 18 patients who needed transfusion. The combination of younger age, intensive chemotherapy and presenting Hb of 12 g/dl or less identified all seven patients who required more than 10 units of blood.

### References

1. Cella D, Dobrez D, Glaspy J. Control of cancer-related anemia with erythropoietic agents: A review of evidence for improved quality of life and clinical outcomes. Ann Oncol 2003;14(4):511–519 [Review].
2. Estrin JT, Schocket L, Kregenow R, Henry DH. A retrospective review of blood transfusions in cancer patients with anaemia. The Oncologist 1999;4:318–324.
3. Lind M, Vernon C, Cruickshank D, Wilkinson P, Littlewood T, Stuart N, Jenkinson C, Grey-Amante P, Doll H, Wild D. The level of haemoglobin in anaemic cancer patients correlates positively with quality of life. Br J Cancer 2002;86(8): 1243–1249.
4. Höckel M and Vaupel P. Biological consequences of tumour hypoxia. Semin Oncol 2001;28(2 Suppl 8):36–41 [Review].
5. Pirollo KF, Bouker KB, Chang EH. Does p53 status influence tumor response to anticancer therapies? Anticancer Drugs 2000;11(6):419–432 [Review].
6. Vaupel P, Kallinowski F, Okunieff P. Blood flow, oxygen and nutrient supply, and metabolic microenvironment of human tumors: A review. Cancer Res 1989;49(23): 6449–6465 [Review].
7. Vaupel P, Thews O, Höckel M. Treatment resistance of solid tumors: Role of hypoxia and anemia. Med Oncol 2001;18(4):243–259.
8. Young SD, Hill RP. Effects of reoxygenation on cells from hypoxic regions of solid tumors: Anticancer drug sensitivity and metastatic potential. J Natl Cancer Inst 1990; 82(5):371–380.
9. Thomlinson RH, Gray LH. The histological structure of some human lung cancers and the possible implications for radiotherapy. Br J Cancer 1955;9(4):539–549.
10. Horsman MR, Overgaard J. The oxygen effect and tumour microenvironment. In: Steel GG, editor. Basic clinical radiobiology. 3rd ed. London: Arnold; 2002.
11. Daly T, Poulsen MG, Denham JW, Peters LJ, Lamb DS, Krawitz H, Hamilton C, Keller J, Tripcony L, Walker Q. The effect of anaemia on efficacy and normal tissue toxicity following radiotherapy for locally advanced squamous cell carcinoma of the head and neck. Radiother Oncol 2003;68(2):113–122.
12. Frommhold H, Guttenberger R, Henke M. The impact of blood hemoglobin content on the outcome of radiotherapy. The Freiburg experience. Strahlenther Onkol 1998;174(Suppl 4):31–34.
13. MacDonald G, Hurman DC. Influence of anaemia in patients with head and neck cancer receiving adjuvant postoperative radiotherapy in the Grampian region. Clin Oncol (R Coll Radiol) 2004;16(1):63–70.
14. Nordsmark M, Overgaard M, Overgaard J. Pretreatment oxygenation predicts radiation response in advanced squamous cell carcinoma of the head and neck. Radiother Oncol 1996;41(1):31–39.
15. Nordsmark M, Alsner J, Keller J, Nielsen OS, Jensen OM, Horsman MR, Overgaard J. Hypoxia in human soft tissue sarcomas: Adverse impact on survival and no association with p53 mutations. Br J Cancer 2001;84(8):1070–1075.
16. Rizzo JD, Lichtin AE, Woolf SH, Seidenfeld J, Bennett CL, Cella D, Djulbegovic B, Goode MJ, Jakubowski AA, Lee SJ, et al. Use of epoetin in patients with cancer: Evidence-based clinical practice guidelines of the American Society of Clinical Oncology and the American Society of Hematology. J Clin Oncol 2002;20(19): 4083–4107.
17. Drug and Therapeutics Bulletin. Epoetins and darbepoetin alfa in malignant disease. Drug and Therapeutics Bulletin 2004;42(3):21–23.
18. Littlewood TJ, Bajetta E, Nortier JW, Vercammen E, Rapoport B. Epoetin Alfa Study Group. Effects of epoetin alfa on hematologic parameters and quality of life in cancer patients receiving nonplatinum chemotherapy: Results of a randomized, double-blind, placebo-controlled trial. J Clin Oncol 2001;19(11):2865–2874.
19. Vansteenkiste J, Tomita D, Rossi G, Pirker R. Darbepoetin alfa in lung cancer patients on chemotherapy: A retrospective comparison of outcomes in patients with mild versus moderate-to-severe anaemia at baseline. Support Care Cancer. 2004;12(4):253–262 [Epub 2004 January 23].
20. Bohlius J, Langensiepen S, Schwarzer G, Seidenfeld J, Piper M, Bennet C, Engert A. Erythropoietin for patients with malignant disease. Cochrane Database Syst Rev 2004;(3):CD003407.
21. Brizel DM, Scully SP, Harrelson JM, Layfield LJ, Bean JM, Prosnitz LR, Dewhirst MW. Tumor oxygenation predicts for the likelihood of distant metastases in human soft tissue sarcoma. Cancer Res 1996;56(5):941–943.
22. Nordsmark M, Hoyer M, Keller J, Nielsen OS, Jensen OM, Overgaard J. The relationship between tumor oxygenation and cell proliferation in human soft tissue sarcomas. Int J Radiat Oncol Biol Phys 1996;35(4):701–708.
23. Stuben G, Pottgen C, Knuhmann K, Schmidt K, Stuschke M, Thews O, Vaupel P. Erythropoietin restores the anemia-induced reduction in radiosensitivity of experimental human tumors in nude mice. Int J Radiat Oncol Biol Phys 2003;55(5):1358–1362.
24. Thews O, Koenig R, Kelleher DK, Kutzner J, Vaupel P. Enhanced radiosensitivity in experimental tumours following erythropoietin treatment of chemotherapy-induced anaemia. Br J Cancer 1998;78(6):752–756.
25. Vansteenkiste J, Pirker R, Massuti B, Barata F, Font A, Fiegl M, Siena S, Gateley J, Tomita D, Colowick AB, et al. Aranesp 980297 Study Group. Double-blind, placebo-controlled, randomized phase III trial of darbepoetin alfa in lung cancer patients receiving chemotherapy. J Natl Cancer Inst 2002;94(16):1211–1220.
26. Glaspy JA, Jadeja JS, Justice G, Kessler J, Richards D, Schwartzberg L, Tchekmedyian NS, Armstrong S, O'Byrne J, Rossi G, et al. Darbepoetin alfa given every 1 or 2 weeks alleviates anaemia associated with cancer chemotherapy. Br J Cancer 2002;87(3):268–276.
27. Littlewood TJ, Nortier J, Rapoport B, Pawlicki M, de Wasch G, Vercammen E, Schuette W, Wils J, Freund M. Epoetin Alfa Study Group. Epoetin alfa corrects anemia and improves quality of life in patients with hematologic malignancies receiving non-platinum chemotherapy. Hematol Oncol 2003;21(4):169–180.
28. Wurnig C, Windhager R, Schwameis E, Kotz R, Zoubek A, Stockenhuber F, Kurz RW. Prevention of chemotherapy-induced anemia by the use of erythropoietin in patients with primary malignant bone tumors (a double-blind, randomized, phase III study). Transfusion 1996;36(2):155–159.
29. Dunst J. The use of epoetin alfa to increase and maintain hemoglobin levels during radiotherapy. Semin Oncol 2001;28(2 Suppl 8):42–48 [Review].
30. Dicato M, Duhem C, Berchem G, Ries F. Clinical benefit from erythropoietin. Curr Opin Oncol 2000;12(4):297–302 [Review].
31. Henry DH, Bowers P, Romano MT, Provenzano R. Epoetin alfa. Clinical evolution of a pleiotropic cytokine. Arch Intern Med 2004;164(3):262–276 [Review].
32. Henry DH. The evolving role of epoetin alfa in cancer therapy. The Oncologist. 2004;9(1):97–107 [Review].
33. Blumberg N, Heal JM, Murphy P, Agarwal MM, Chuang C. Association between transfusion of whole blood and recurrence of cancer. Br Med J (Clin Res Ed) 1986;293(6546):530–533.
34. McClinton S, Moffat LE, Scott S, Urbaniak SJ, Kerridge DF. Blood transfusion and survival following surgery for prostatic carcinoma. Br J Surg 1990;77(2):140–142.
35. Leyland-Jones B. BEST Investigators and Study Group. Breast cancer trial with erythropoietin terminated unexpectedly. Lancet Oncol 2003;4(8):459–460.
36. Henke M, Laszig R, Rube C, Schafer U, Haase KD, Schilcher B, Mose S, Beer KT, Burger U, Dougherty C, Frommhold H. Erythropoietin to treat head and neck cancer patients with anaemia undergoing radiotherapy: Randomised, double-blind, placebo-controlled trial. Lancet 2003;362(9392):1255–1260.
37. Rosenzweig MQ, Bender CM, Lucke JP, Yasko JM, Brufsky AM. The decision to prematurely terminate a trial of R-HuEPO due to thrombotic events. J Pain Symptom Manage 2004;27(2):185–190.
38. Walker RH. Special report: Transfusion risks. Am J Clin Pathol 1987;88(3):374–378.
39. Varney SJ, Guest J F. The annual cost of blood transfusions in the UK. Transfus Med 2003;13:2005–2218.
40. Barosi G, Marchetti M, Liberato NL. Cost-effectiveness of recombinant human erythropoietin in the prevention of chemotherapy-induced anaemia. Br J Cancer 1998;78(6):781–787.
41. Kavanagh BD, Fischer BA 4th, Segreti EM, Wheelock JB, Boardman C, Roseff SD, Cardinale RM, Benedict SH, Goram AL. Cost analysis of erythropoietin versus blood transfusions for cervical cancer patients receiving chemoradiotherapy. Int J Radiat Oncol Biol Phys 2001; 51(2):435–441.

